# Leçons de Gynécologie Opératoire
*Par MM. Vulliet et Lutaud. Paris: (A. Maloine, 1890.) Pp. 500. Edition deuxieme.


**Published:** 1890-05-24

**Authors:** 


### THE PRACTITIONER'S BOOKSHELF.

[Books for Review should be sent to The Editor, The Lodge, Porchester Square, W.]

### LEÇONS DE GYNÉCOLOGIE OPÉRATOIRE*.

These twenty-five lessons constitute a complete course of operative gynecological lectures. We have looked through the volume from the preliminary chapter to the end, and have found it well up to date. The authors have not only done much original work themselves but have kept abreast of the times, and we notice that the names of authors of all countries and their methods of treatment are repeatedly quoted, often fully. In speaking of the method of Apostoli for the cure of fibroids it is stated that though several successful results ensued from its use, yet the amelioration produced was not so quick as when the faradic currents generated by the small apparatus of Gaiffe were used. Page 189. A considerable number of pessaries are figured, and the methods of placing some of them. Hodge's pessary for retroversion and Graily Hewitt's for anteversion are both recognised as most valuable instruments. Professor Vulliet describes his ingenious instrument for preventing prolapsus; the action is practically that of a Hodge, but there is a double curve in the anterior portion, which tends to produce more support to the vesical portion of the vaginal wall. Lawson Tait's method of restoring the perinæum after rupture is described *in extenso,* with plates of the various steps of the operation. We note that the book contains no less than 200 illustrations. Some of these are remarkably good, but others are too blurred to make out the dotted lines and letters of explanation. As the work is got up merely stitched and in paper covers, like so many French text-books, and as the cost is probably much less than a work of similar size published in this country, we must not be too particular with regard to this point. The authors are quite aware of the importance of antiseptics in gynecology, and the various methods of their use are noted in full. We find in this work a very full description of the treatment of uterine cancer. The authors state that Freund's operation, laparo-hysterectomy is, at present, generally abandoned and ought only to be used when it is found that the inferior aperture is too small to extract the uterine mass after it has been separated from below. Professor Vulliet in suitable cases uses the actual cautery; he figures a lamp for heating the irons on the principle of the glass blower's apparatus, so that a number of cauteries can be heated at once. His method is to dilate the uterus and then by a combination of excision and cauterisation to clear away the carcinomatous growth. His statistics give an encouraging outlook to this form of operation. There is also a well-illustrated chapter on uterine massage, which method of cure is still tentatively before the profession. In conclusion, we consider this a most valuable work as giving a clear account of the state of this special branch of medicine in the schools of Geneva (Vulliet) and Paris (Lutaud). Certain chapters doubtless are more fully written than in similar works published in this country; this, we take it, is because the racial habits of thought of our friends and neighbours across the sea are different from ours. We notice but few mistakes in the spelling of English names. University College, London, is, however called the College of the University of London, which it was originally, but has long ceased to be. There is a full index to the lessons and a list of the plates.

*Par MM. Vulliet et Lutaud. Paris: (A. Maloine, 1890.) Pp. 500. Edition deuxième.

